# Peripheral Blood TIM-3 Positive NK and CD8+ T Cells throughout Pregnancy: TIM-3/Galectin-9 Interaction and Its Possible Role during Pregnancy

**DOI:** 10.1371/journal.pone.0092371

**Published:** 2014-03-20

**Authors:** Matyas Meggyes, Eva Miko, Beata Polgar, Barbara Bogar, Balint Farkas, Zsolt Illes, Laszlo Szereday

**Affiliations:** 1 University of Pecs, Clinical Centre, Department of Medical Microbiology and Immunology, Pecs, Hungary; 2 Janos Szentagothai Research Centre, Pecs, Hungary; 3 University of Pecs, Clinical Centre, Department of Obstetrics and Gynaecology, Pecs, Hungary; 4 Odense University Hospital, Department of Neurology, Odense, Denmark; 5 University of Southern Denmark, Institute of Clinical Research, Odense, Denmark; INSERM-Université Paris-Sud, France

## Abstract

**Problem:**

The T-cell immunoglobulin and mucin domain (TIM) family is a relatively newly described group of molecules with a conserved structure and important immunological functions. Identification of Galectin-9 as a ligand for TIM-3 has established the Galectin-9/TIM-3 pathway as an important negative regulator of Th1 immunity and tolerance induction. Data about the TIM-3/Gal-9 pathway in the pathogenesis of human diseases is emerging, but their possible role during human pregnancy is not precisely known. The aim of our study was to investigate the number, phenotype and functional activity of TIM-3+ peripheral blood mononuclear cells during healthy human pregnancy.

**Methods of Study:**

57 healthy pregnant women [first trimester (n = 16); second trimester (n = 19); third trimester (n = 22)] and 30 non-pregnant controls were enrolled in the study. We measured the surface expression of TIM-3 by cytotoxic T cells, NK cells and NK cell subsets as well as Galectin-9 expression by regulatory T cells by flow cytometry. We analyzed the cytokine production and cytotoxicity of TIM3+ and TIM3- CD8 T and NK cells obtained from non-pregnant and healthy pregnant women at different stages of pregnancy by flow cytometry. Serum Galectin-9 levels were measured by ELISA.

**Results:**

Our results show that the numbers of peripheral NK and cytotoxic T cells and their TIM-3 expression do not change between the first, second and third trimesters of pregnancy. Compared to non-pregnant individuals, regulatory T cells show higher level of Galectin-9 expression as pregnancy proceeds, which is in line with the level of Galectin-9 in the patients sera. Cytotoxic T cells, NK cells and NK cell subsets expressing TIM-3 molecule show altered cytokine production and cytotoxicity during pregnancy compared to non-pregnant individuals.

**Conclusion:**

Our results indicate that Galectin-9 expressing regulatory T cells, TIM-3+ cytotoxic T cells and NK cells could play an important role in the maintenance of healthy pregnancy.

## Introduction

During healthy pregnancy, the maternal immune system has to be altered to enable survival of the semi-allogeneic fetus. Pregnancy is an ideal condition to study active immunotolerance. During pregnancy the fetus will not be attacked or rejected by the maternal immune system but rather successfully accepted by the mother. Precise immunoregulation of the maternal immune system is critical for normal pregnancy and fetal development.

For many years Th1/Th2 hypothesis has provided a useful framework for studies of the immunology of pregnancy. However, the findings that pregnancy itself is an inflammatory state has led to a revision of this hypothesis and now it is apparent that both arms of the immune response are intensified during healthy pregnancy, but with a stronger bias towards Th2 than Th1 responses [1]–[3]. The participation of NK and NKT cells in the Th1/Th2 shifts of pregnancy suggests a dominant role of the innate rather than the adaptive immune system [4]. The Th1/Th2 paradigm has recently been reconstituted to include a third population of T helper cells that produce IL-17, therefore these cells are designated as Th17 cells [5]. This Th2 cytokine polarization occurs both at systemic level and at the fetal-maternal interface, [6] and the cause behind this cytokine shift are not clearly defined.

Pregnancy as a physiological condition includes the altered ratio and function of different lymphocytes subpopulations compared to non-pregnant status. Therefore it is important to investigate and understand the immune regulatory mechanism behind these immunological changes.

The immunoglobulin superfamily member T-cell immunoglobulin mucin 3 (TIM-3) was first discovered in 2002 on interferon IFN-γ producing CD4+ (Th1) and on CD8+ T cytotoxic cells (Tc) [7]. TIM-3 expression was verified in a variety of immune cells, including Th1, Th17, NK cells, NKT cells, Tregs, and also on antigen presenting immune cells such as dendritic cells and monocytes [8]. TIM-3 molecule has been implicated in both activation and inhibition of immune responses [9], [10], but its *in vivo* function have remained unknown. Expression of TIM-3 on Th1 cells provides a key checkpoint that serves to dampen proinflammatory Th1-dependent T-cell responses and may contribute to the maintenance of pregnancy. In line with this, Chabtini et al. examined the TIM-3-expression on innate immune cells by using an allogeneic mouse model of pregnancy and indicated their possible role in the regulation of tolerance at the fetomaternal interface [11]. The only human study presented that TIM-3 is up-regulated by monocytes in peripheral blood of pregnant women indicate that abnormal TIM-3 expression might be related to the loss of pregnancy [8].

Galectin-9 (Gal-9) is a member of a family of evolutionary conversed endogenous lectins and is characterized by the presence of two carbonhydrate recognition domains with affinity for β-galactoside [12]. Many studies examined the role of Gal-9 in immunological contexts, which can influence the immune system in different ways, either by exacerbating the inflammatory process [13] or by acting as an anti-inflammatory agent [14].

Among several identified receptors of Gal-9, TIM-3 has been studied most extensively. There is evidence that engagement of TIM-3 by its ligand Gal-9 leads to the death of Th1 and Th17 cells, furthermore influences the ability to induce T cell tolerance in both mice and humans [15]–[17]. Thus, engagement of TIM-3 by Gal-9 may function as a negative regulator, abrogating Th1- and Th17 driven immune responses and may modulate the Th1/Th2 balance.

Recently, human Gal-9+ Th cells were identified expressing Gal-9 on their surface and secreting Gal-9 upon TCR stimulation resulting in the regulation of Th17/Treg development [18]. Moreover it was shown that IL-6 abrogates the increase of Gal-9+ Th cells *in vitro*, and indicates that neutralization of IL-6 may be a strategy for increasing Gal-9+ Th cells in order to ameliorate Th1/Th17-skewed immunity.

Supporting a reproductive role for Gal-9, M von Wolff et al. and Popovici et al. found that human endometrium and decidua express Gal-9 and it’s strong expression suggest a potential role during implantation [19]–[21]. In this regard, it is possible that TIM-3/Gal-9 interaction could play an important role in the regulation of maternal immune tolerance toward the fetus and may be a potent regulator of the adaptive and innate immune responses. Although data about the TIM-3/Gal-9 pathway in the pathogenesis of human diseases is emerging their possible role during human pregnancy is not precisely known.

Therefore our aim was to investigate the expression of TIM-3 and Gal-9 on different T and NK cell subsets throughout pregnancy and in non-pregnant women, and to determine the pregnancy related phenotypic changes of peripheral blood mononuclear cells. We suppose that their interaction plays a significant role in maintenance of healthy pregnancy.

## Materials and Methods

### Patients

To study Gal-9 and TIM-3 expression throughout pregnancy, 16 healthy women were recruited in the first trimester, 19 in the second, 22 in the third trimester and 30 non-pregnant healthy women were included in this study (Table I). None of the women had any significant medical history, current or recent illnesses, or were taking medications.

### Ethics statement

Written informed consent was obtained from all participants. The study protocol conforms to the ethical guidelines of the 1975 Declaration of Helsinki as reflected in a priori approval by the Regional Ethical Committee at the Faculty of Medicine, University of Pécs.

### Lymphocyte separation, cryopreservation and thawing

Peripheral blood mononuclear cells (PBMC) were isolated from heparinized venous blood on Ficoll-Paque gradient (GE Healthcare Life Sciences, USA). After washing in RPMI 1640 medium (Lonza, Switzerland) the cells were counted centrifuged and resuspended in human AB serum (Lonza, Switzerland) containing 10% DMSO for cryoprotection. Cells were aliquoted in cryovials and stored in a –80°C mechanical freezer. Thawing was carried out on the day of fluorescent cell labeling as quickly as possible in 37°C water bath and DMSO (Sigma-Aldrich, Hungary) was washed out twice in RPMI 1640 medium.

### Antibodies

Freshly thawed PBMC were used for surface and intracellular staining and analysis. The following monoclonal antibodies were used: fluorescein isothiocyanate (FITC)-conjugated anti-human CD3 (BD Pharmingen, USA), FITC-conjugated anti-human CD4 (BD Pharmingen, USA), FITC-conjugated anti-human CD8 (BD Pharmingen, USA), FITC-conjugated anti-human CD107a (BD Pharmingen, USA), phycoerythrin (PE)-conjugated anti-human Galectin-9 (Biolegend, USA), PE-conjugated anti-human TIM-3 (R and D Systems, USA), allophycocyanin (APC)-conjugated anti-human CD56 (BD Pharmingen, USA), APC-conjugated anti-human CD8 (BD Pharmingen, USA), APC-conjugated anti-human TIM-3 (R and D Systems, USA) APC-conjugated anti-human FoxP3 (eBioscience, USA). Control antibodies included isotype-matched FITC-conjugated, PE-conjugated and APC-conjugated mouse antibodies (all from BD-Pharmingen, USA).

### Labeling of lymphocytes and flow cytometric analysis

Cryopreserved cells were quickly thawed and washed with PBS (Lonza, Switzerland) to remove DMSO. 10^6^ PBMC in 100 μl PBS/tube was incubated for 30 minutes at room temperature with the fluorochrome-labeled monoclonal antibodies. After washing, the cells were resuspended in 300 μl PBS containing 1% paraformaldehyde, and stored at 4°C in dark until FACS analysis. Labeled cells were analyzed with a FACSCalibur flow cytometer (BD Immunocytometry Systems, Erembodegen, Belgium) equipped with the CellQuest software program (BD Biosciences, San Diego, CA, USA) for data acquisition and analysis.

### CD107a cytotoxicity assay

To determine CD107a surface expression by cytotoxic T cells and NK cells, PBMC were incubated for 4 h at 37°C in an atmosphere containing 5% CO_2_ in the presence of FITC-conjugated anti-human CD107a monoclonal antibody in RPMI 1640 medium containing 10% fetal bovine serum, penicillin and streptomycin (Lonza, Switzerland), ionomycin (Sigma–Aldrich, Hungary) and phorbol myristate acetate (Sigma–Aldrich, Hungary). After stimulation the cells were washed and resuspended in PBS then stained with antibodies to Tc cell and NK cell markers (APC-conjugated anti-human CD8 or APC-conjugated anti-human CD56) together with PE-conjugated anti-human TIM-3 antibody for 30 min at room temperature. The cells were washed in PBS, fixed with 1% paraformaldehyde and evaluated by FACSCalibur flow cytometer (BD Immunocytometry Systems, Erembodegen, Belgium) equipped with the CellQuest software program (BD Biosciences, San Diego, CA, USA) for data acquisition and analysis.

### FoxP3 staining

After surface labeling intracellular staining of FoxP3 was performed using the FoxP3 Staining Buffer Set (eBioscience, USA) according to the manufacture’s protocol. Briefly, cells were permeabilized in 1 ml fixation/permeabilization buffer (Concentrate/Diluent 1:4) at 4°C for 1 h. Then the samples were washed twice in buffer and stained with the APC-conjugated anti-human FoxP3 monoclonal antibody at 4°C for 1 hour. Flow cytometric analysis was performed on FACSCalibur flow cytometer (BD Immunocytometry Systems, Erembodegen, Belgium) equipped with the CellQuest software program (BD Biosciences, San Diego, CA, USA) for data acquisition and analysis.

### MACS separation

PBMC were separated from heparinized venous blood on Ficoll-Paque gradient. For the MACS separation, up to 2×10^8^ total cell were centrifuged at 1500 rpm for 10 min in cold MACS buffer (PBS with 5% FCS). The samples were resuspended in 2ml cold MACS buffer then applied to pre-separation filters to remove cell clumps and centrifuged at 1500 rpm for 10 min in cold MACS buffer. Magnetic bead-conjugated anti-human CD56 antibody (MACS, Miltenyi Biotech, Germany) (20 μl/10^7^ cell) was used to label NK cells for 15 min on ice. After labeling the cell suspensions were centrifuged at 1200 rpm for 10 min in cold MACS buffer. Labeled cells were resuspended in 500 μl of cold MACS buffer and put a magnetic separation (MS) column (Miltenyi Biotec, Germany), which was freshly equilibrated with 500 μl cold MACS buffer. Unlabeled CD56− cells passed through the column, whereas the CD56+ cells remained in the column due to the magnetic field. After triple washing with 500 μl of cold MACS buffer, the column was removed from the magnetic field and the CD56+ fraction were eluted with 1 ml cold MACS buffer using a plunger. The unlabeled cell fractions (CD56−) were centrifuged at 1500 rpm for 10 min, resuspended in an appropriate volume of cold MACS buffer and labeled with a human CD8 (MACS, Miltenyi Biotech, Germany) magnetic bead-conjugated antibody (20 μl/10^7^) cell.

The separated CD56+ and CD8+ cells were washed with PBS at 1500 rpm for 10 min. The CD56+ cell pellets were then incubated with of FITC-conjugated anti-human CD3, PE-conjugated anti-human TIM-3 and APC-conjugated anti-human CD56 for 30 min at room temperature in dark. The CD8+ cell pellets were incubated with PE-conjugated anti-human TIM-3 and APC-conjugated anti-human CD8 for 30 min at room temperature in dark. The cells were washed with PBS at 2000 rpm for 5 min and resuspended in 400 μl PBS for sorting.

### Cell sorting

The MACS separated and labelled cells were sorted using a BD FACS ARIA II with a BD FACS Diva V6 software (BD Biosciences, USA). NK cells and CD8 cells were recognized based on scatter characteristics and expression of different markers. CD3-TIM-3-CD56dim, CD3-TIM-3+CD56dim CD3-TIM-3-CD56bright, CD3-TIM-3+CD56bright, CD8+TIM-3- and CD8+TIM-3+ cells were separated. After sorting, the purity of the cell fraction was measured on the BD FACS ARIA (BD Immunocytometry Systems, Erembodegen, Belgium) equipped with a 70 μm nozzle. Purity was greater than 95%.

Sorted cells were collected in 300 ml RPMI 1640 medium containing 10% fetal bovine serum. After sorting the cells were washed with PBS and centrifuged at 1500 rpm for 10 min. The supernatants were aspirated then the remaining cells were incubated overnight at 37°C in the presence of RPMI 1640 medium containing 10% fetal bovine serum, penicillin and streptomycin (Lonza, Switzerland), ionomycin (Sigma–Aldrich, Hungary) and phorbol myristate acetate (Sigma–Aldrich). After stimulation the cells were centrifuged at 2000 rpm for 10 minutes. The supernatants were collected, aliquoted and stored at –80°C in a mechanical freezer for further cytokine analysis.

### Cytometric Bead Array (CBA)

Concentration of cytokines from supernatants were determined using a Human Th1/Th2/Th17 CBA kit (BD Biosciences, USA) which allowed for the simultaneous detection of IL-2, IL-4, IL-6, IL-10, TNF-α, IFN-γ and IL-17A. Aliquoted samples were thawed and CBA analysis performed according to the manufacturer’s protocol. Briefly, beads coated with capture antibodies were mixed. 50 μl of the capture bead mixture was added to 50 μl of sample. To these sample-bead compounds, 50 μl of phycoerythrin conjugated detection antibody was added and this mixture was incubated for 3 h in dark at room temperature. The samples then were washed with 1 ml of wash buffer at 1100 rpm for 5 min and the pellets were resuspended in 300 μl wash buffer. Cytokine standards were serially diluted to facilitate the construction of calibration curves necessary for determining protein concentrations of test samples. Flow cytometric analysis was performed on a BD FACSCanto II (BD Immunocytometry Systems, Erembodegen, Belgium) with BD FACS DIVA software V6 and data were analyzed with FCS Express V3 software.

### Galectin-9 ELISA

Serum samples were collected from healthy non-pregnant individuals and from the 1^st^, the 2^nd^ and the 3r^d^ trimester group of gestation. 10ml venous blood was taken to disposable sterile test tube and centrifuged at 2000 rpm for 10 minutes. Serum samples then transferred in 1ml aliquots into cryovials and stored frozen at –80°C until analysis.

Serum Gal-9 levels were measured by ELISA in 26 non-pregnant women and in 10 healthy women in each trimester of pregnancy.

The serum Gal-9 concentration was determined by a quantitative sandwich enzyme immunoassay according to the manufacturer’s protocol (BLUEGENE GAL9 ELISA kit, AMS Biotechnology, UK). Briefly, 50 μl/well of standard and human serum samples obtained from healthy pregnant woman and normal controls were added to 96 well microplate pre-coated with monoclonal antibody specific for Gal-9 protein. Then 100 μl of enzyme conjugate (horseradish peroxidase-conjugated polyclonal anti-Gal-9) was added to each well, mixed thoroughly and incubated for 1 hour at 37°C. Then the wells of the microtiter plate were washed 5 times with 200 μl of Wash buffer. For color development 50 μl of Substrate A and 50 μl of Substrate B were added to each well, and incubated for 10–15 minutes at 37°C in the dark. Finally 50 μl/well of Stop solution was added to the wells and mixed with gentle tapping to terminate the reaction. The optical density was measured at 450nm with FluoSTAR Optima (BMG Labtech, Germany) microplate spectrophotometer and the Gal-9 protein concentration was determined with Optima 2.10 R2 built-in data calculator software.

### Statistical analysis

Statistical analysis was performed using statistical software SPSS version 20. package. Multiple comparisons were made using one-way ANOVA with Bonferroni correction. Differences were considered significant if the p value was equal to or less than 0.05.

## Results

### 1. Phenotype analysis of peripheral blood mononuclear cells throughout pregnancy and in non-pregnant women

We investigated the percentage of CD3+ T cells, CD4+, CD8+ T cell subpopulations, NK cells, NKT cells and Gal-9+ Th cells in the peripheral blood of normal pregnant women during each trimester of pregnancy and in non-pregnant women.

In this study investigating healthy pregnant women, CD3+, CD4+, CD8+ T cell, NKT cells, NK cell and NK subpopulation levels were not significantly changed during first, second and third trimester even to compared to non-pregnant women. The frequency of NK cells and CD56dim NK cells throughout pregnancy was lower and the frequency of CD56bright cells was higher than in non-pregnant women but these results did not reach the level of significance (Table II).

Gal-9 staining revealed the existence of surface Gal-9-expressing CD4 T cells (Gal-9+ Th cells) [18]. The frequency of these cells were approximately 1% in non-pregnant women and we detected an increased frequency throughout pregnancy reaching 2,39 % in the third trimester (Table II). The frequency of Gal-9 Th cells in the third trimester was significantly higher than in non-pregnant women, as well as women in the first and second trimester (Table II).

### 2. Differential TIM-3 expression by peripheral blood mononuclear cell subsets throughout pregnancy and in non-pregnant women

We measured the surface expression of TIM-3 by Th cells, Tc cells, NK cells, NK cell subsets and NKT cells by flow cytometry.

**Figure 1:**
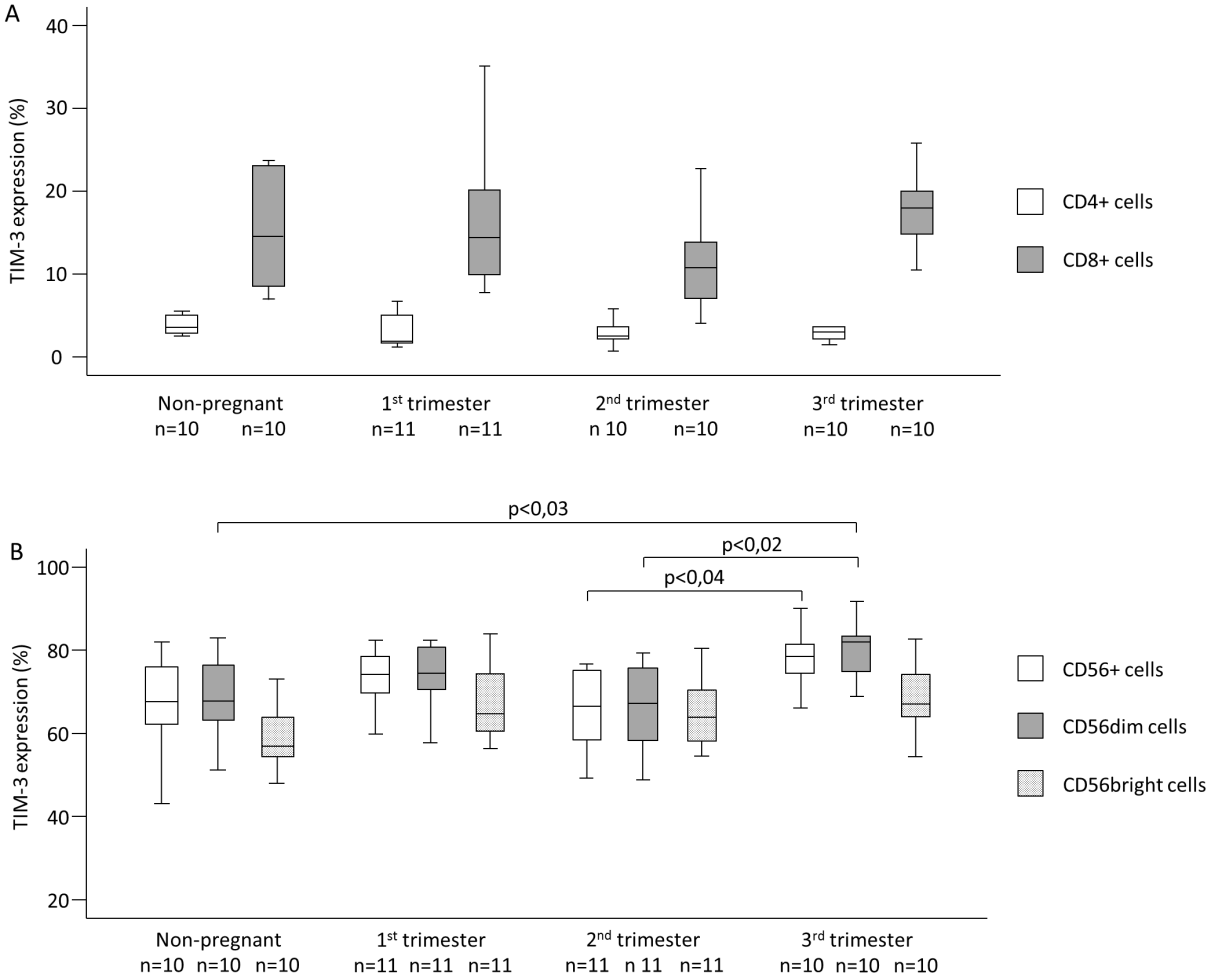
TIM-3 expression by peripheral blood mononuclear cell subsets throughout pregnancy and in non-pregnant women. The expression of TIM-3 by Th cells, Tc cells, NK cells and NK cell subsets in the peripheral blood of normal pregnant women during each trimester of pregnancy and in non-pregnant women. The solid bars represent medians, the boxes indicate the interquartile ranges and the lines show the most extreme observations. Differences were considered statistically significant for P-values ≤0.05.

TIM-3 expression by the CD4 T cells showed no significant difference between any investigated groups (Fig. 1A). Investigating TIM-3 expression by CD8+ T cells we found a decrease in the 2nd trimester compared to other trimesters and to the samples from the non-pregnant group, however this change did not reach the level of statistical significance (Fig. 1A). Furthermore TIM-3 expression was significantly increased by NK cells in samples from the 3rd trimester compared to the samples in the 2nd trimester (65,91±2,91 vs. 78,07±2,23) (Fig. 1B). Analyzing the NK cell subsets, the TIM-3 expression by CD56dim NK cells was significantly increased in samples from the third trimester compared to the samples from the second trimester and from non-pregnant women (66,01±3,20 vs. 79,97±2,29; 67,18±3,88 vs. 79,97±2,29). In the case of CD56bright cells we only found an increasing tendency during the three trimesters compared to non-pregnant women, but these results were not statistically significant (Fig. 1B). TIM-3 expression by NKT cells did not show any significant difference between any investigated groups (data not shown).

**Table 1:** Characteristics of Non-pregnant, 1^st^, 2^nd^ and 3^rd^ trimester pregnant women.

	Non-pregnant	1^st^ trimester	2^nd^ trimester	3^rd^ trimester
**No. of patients**	30	16	19	22
**Age (years)**	33 (19–44)	31 (27–35)	32 (26–45)	33 (28–43)
**Gestation age at sampling (weeks)**	-	12 (11–16)	26 (24–28)	36 (35–38)
**Gestation age at birth (weeks)**	-	38,8 (35–41)	38,0 (33–41)	39,0 (37–41)
**Gravidity**	1,5	1,2	1,9	1,7
**Parity**	1,0	0,4	0,7	0,5

Data are shown as mean (range). Statistical comparisons were made by using the ANOVA tests. Results did not differ significantly between study groups.

### 3. Downregulation of proinflammatory cytokine production by peripheral CD8+ T, CD56dim and CD56bright NK cells expressing TIM-3

Th1, Th2 and Th17 cytokines were analyzed by CBA system, where IL-4, IL-6 and IL-1 cytokines were under the detectable level.

CD8+ T cells expressing TIM-3 produced significantly lower level of proinflammatory (IL-2, TNF-α and IFN-γ) and Th17 cytokines compared to TIM-3 negative counterparts in the first and third trimester of pregnancy and in healthy non-pregnant controls (Table III).

IL-2 cytokine production by TIM-3 positive CD56dim NK cells was significantly lower compared to TIM-3 negative CD56dim NK cells in the first and second trimester of pregnancy (Table III).

In the second trimester of pregnancy IFN-γ cytokine production by TIM-3 positive CD56bright NK cells was significantly higher compared to TIM-3 negative CD56bright NK cells (Table III).

### 4. Altered cytotoxic activity of TIM-3 expressing peripheral CD8+ T cells, NK cells and NK cell subsets throughout pregnancy and in non-pregnant women

**Figure 2:**
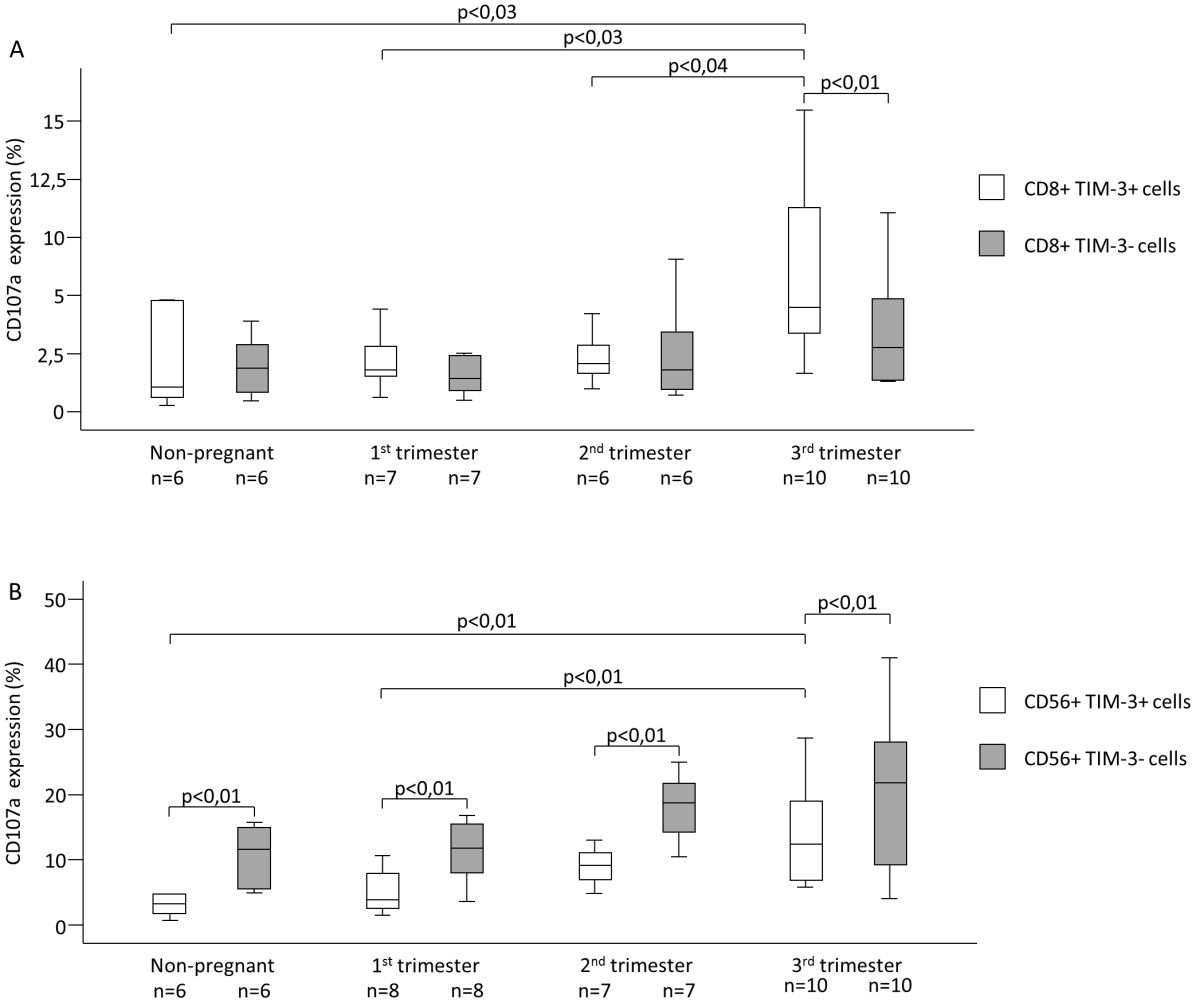
CD107a expression by peripheral blood mononuclear cell subsets throughout pregnancy and in non-pregnant women. The expression of CD107a by TIM-3 negative and TIM-3 positive cytotoxic T cells and NK cells in the peripheral blood of normal pregnant women during each trimester of pregnancy and in non-pregnant women. The solid bars represent medians, the boxes indicate the interquartile ranges and the lines show the most extreme observations. Differences were considered statistically significant for P-values ≤0.05.

Investigating the cytotoxic activity of TIM-3+ CD8+ Tc cells during pregnancy, we found that CD107a expression was significantly higher in samples from 3rd trimester compared to 1st and 2nd trimester and non-pregnant women (6,32±1,19 vs. 2,22±0,50; 6,32±1,19 vs. 2,32±0,46; 6,32±1,19 vs. 2,11±0,86) (Fig. 2A). CD8+ T cells expressing TIM-3 in the third trimester of pregnancy showed significantly increased CD107a expression compared to TIM-3 negative CD8+ T cells (Fig. 2A).

**Table 2:** Peripheral blood mononuclear cell phenotype characteristics throughout pregnancy and in non-pregnant women.

	Non-pregnant	1^st^ trimester	2^nd^ trimester	3^rd^ trimester	P-value
**CD3+ T cells**	**66,64**±2,23	**65,10**±3,27	**69,43**±3,33	**67,74**±1,34	NS
**CD4+ T cells**	**44,04**±2,32	**39,92**±2,28	**45,68**±3,47	**39,03**±2,60	NS
**CD8+ T cells**	**32,02**±1,51	**35,25**±1,53	**28,98**±2,67	**34,31**±2,41	NS
**CD8+ TIM-3+ T cells**	**5,59**±0,83	**6,0**±0,89	**4,36**±0,88	**5,97**±0,50	NS
**CD3- CD56+ cells**	**13,65**±1,38	**12,31**±1,85	**10,69**±1,74	**11,47**±1,53	NS
**CD3- CD56dim cells**	**12,72**±1,31	**10,56**±1,75	**9,29**±1,69	**9,74**±1,39	NS
**CD3- CD56bright cells**	**1,03**±0,24	**1,80**±0,29	**1,44**±0,30	**1,76**±0,24	NS
**CD3- CD56+ TIM-3+ cells**	**9,61**±1,41	**9,16**±1,53	**7,34**±1,30	**9,05**±1,30	NS
**CD3- CD56dim TIM-3+ cells**	**9,02**±1,38	**8,10**±1,42	**6,49**±1,25	**8,07**±1,23	NS
**CD3- CD56bright TIM-3+**	**0,61**±0,18	**1,11**±0,22	**0,93**±0,24	**1,11**±0,17	NS
**CD3+ CD56+ cells**	**4,08**±0,75	**6,37**±1,21	**4,70**±0,98	**5,46**±1,26	NS
**Gal-9+ Th cells**	**0,97**±0,13	**0,66**±0,09	**1,17**±0,3	**2,39**±0,49	**3^rd^ vs. NP p<0,01 3^rd^ vs. 1^st^ p<0,01 3^rd^ vs. 2^nd^ p<0,03**

Statistical comparisons were made by using the ANOVA tests. The results were expressed as the mean value±standard error of the mean (SEM). Differences were considered significant when the value of p was equal to or less than 0.05. NS  =  not statistically significant.

**Figure 3:**
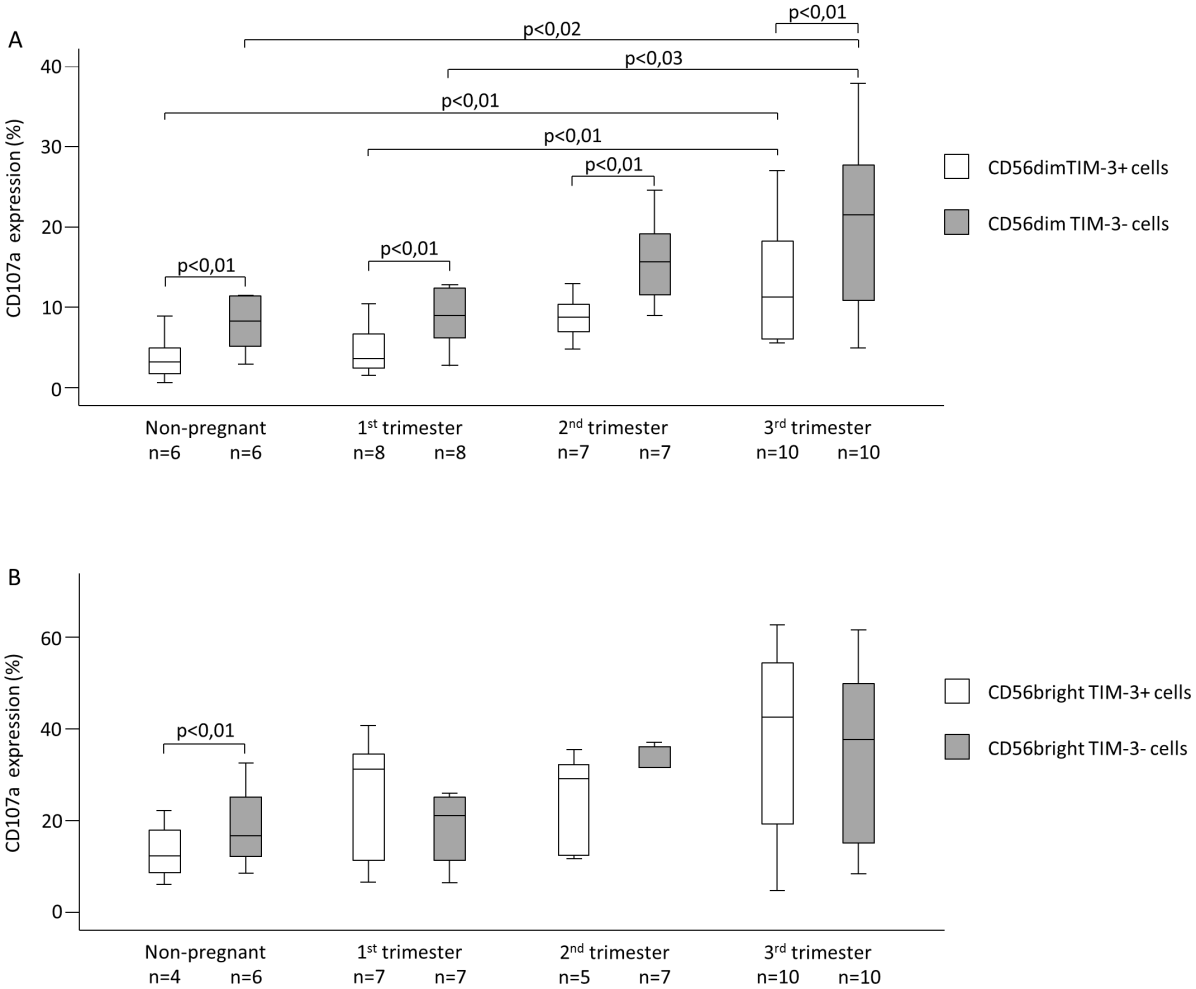
CD107a expression by peripheral blood mononuclear cell subsets throughout pregnancy and in non-pregnant women. The expression of CD107a by TIM-3 negative and TIM-3 positive CD56dim and CD56bright cells in the peripheral blood of normal pregnant women during each trimester of pregnancy and in non-pregnant women. The solid bars represent medians, the boxes indicate the interquartile ranges and the lines show the most extreme observations. Differences were considered statistically significant for P-values ≤0.05.

Furthermore TIM-3 positive NK cells and NKdim subset showed similar CD107a expression pattern, where the cytotoxic activity was significantly increased in samples from 3rd trimester compared to the non-pregnant group and 1st trimester group (NK cells: 3,83±1,27 vs. 17,38±3,29; 5,11±1,17 vs. 17,38±3,29 (Fig. 2B); NKdim cells: 3,76±1,23 vs. 15,73±3,19; 4,69±1,07 vs. 15,73±3,19 (Fig. 3A)). Analyzing CD107a expression by the NKbright subpopulation showed no significant differences (Fig. 3B). Interestingly cytotoxic activity of TIM-3 positive NK cell and NKdim cell were significantly lower in non-pregnant women and in all trimesters compared to TIM-3 negative counterparts (Fig. 2B and 3A).

**Table 3:** Cytokine production by TIM-3 positive and negative CD8+ T, CD56dim and CD56bright NK cells.

		Non-pregnant			1^st^ trimester			2^nd^ trimester			3^rd^ trimester		
	*pg/ml*	TIM-3+	TIM-3-	p	TIM-3+	TIM-3-	p	TIM-3+	TIM-3-	p	TIM-3+	TIM-3-	p
**CD56**	IL-2	9,18	117,18	NS	9,53	62,95	**<0,04**	9,25	17,17	**<0,01**	9,23	80,71	NS
**dim**	TNF-α	1625,01	2157,81	NS	1820,63	2278,48	NS	104,75**	793,57	NS	439,75**	403,62	NS
**cells**	IFN-γ	2010,29	2172,1	NS	3095,85	3516,78	NS	218,03**	1095,87	NS	816,23***	696,77^$^	NS
	IL-17	<min	<min	NS	<min	<min	NS	<min	<min	NS	<min	<min	NS
**CD56**	IL-2	20,97	114,27	NS	13,25	39,99	NS	14	12,78	NS	12,93	50,89	NS
**dim**	TNF-α	1211,37	1786,06	NS	1036,76	1315,13	NS	641,82	461,83	NS	1188,97	1046,67	NS
**cells**	IFN-γ	1961,81	3513,19	NS	2656,23	2543,13	NS	1632,29	979,18	**<0,04**	1894,9	2136,18	NS
	IL-17	<min	<min	NS	<min	<min	NS	<min	<min	NS	<min	<min	NS
	IL-2	3076,06	6400,32	**<0,05**	2463,73	7812,88	**<0,01**	687,87	1276,9*	NS	2587,58	7471,57	**<0,03**
**CD8**	TNF-α	1385,92	6064,46	**<0,01**	1217,89	6654,4	**<0,01**	356,7	1468,89^$^	NS	861,61	3427,65	**<0,01**
**T cells**	IFN-γ	3455,09	5750,34	NS	4223,87	6306,06	**<0,05**	908,5^$$$^	2093^$$^	NS	2364,49	4851,96	**<0,04**
	IL-17	17,33	345,2	**<0,04**	16,93	247,9	**<0,05**	9,83	11,49	NS	20,83	257,01	**<0,04**

Statistical comparisons were made by using the ANOVA tests. The results were expressed as the mean value. Differences were considered significant when the value of p was equal to or less than 0.05. NS  =  not statistically significant.

*Sig. from NP;1st; 3rd (p<0,01).

**Sig. from NP;1st (p<0,01).

***Sig. from NP (p<0,05);1st (p<0,01).

$Sig. from 1st (p<0,01).

$$Sig. from 1st (p<0,04).

$$$Sig. from NP (p<0,03);1st (p<0,05).

### 5. Circulating Galectin-9 levels throughout pregnancy and in non-pregnant women

**Figure 4:**
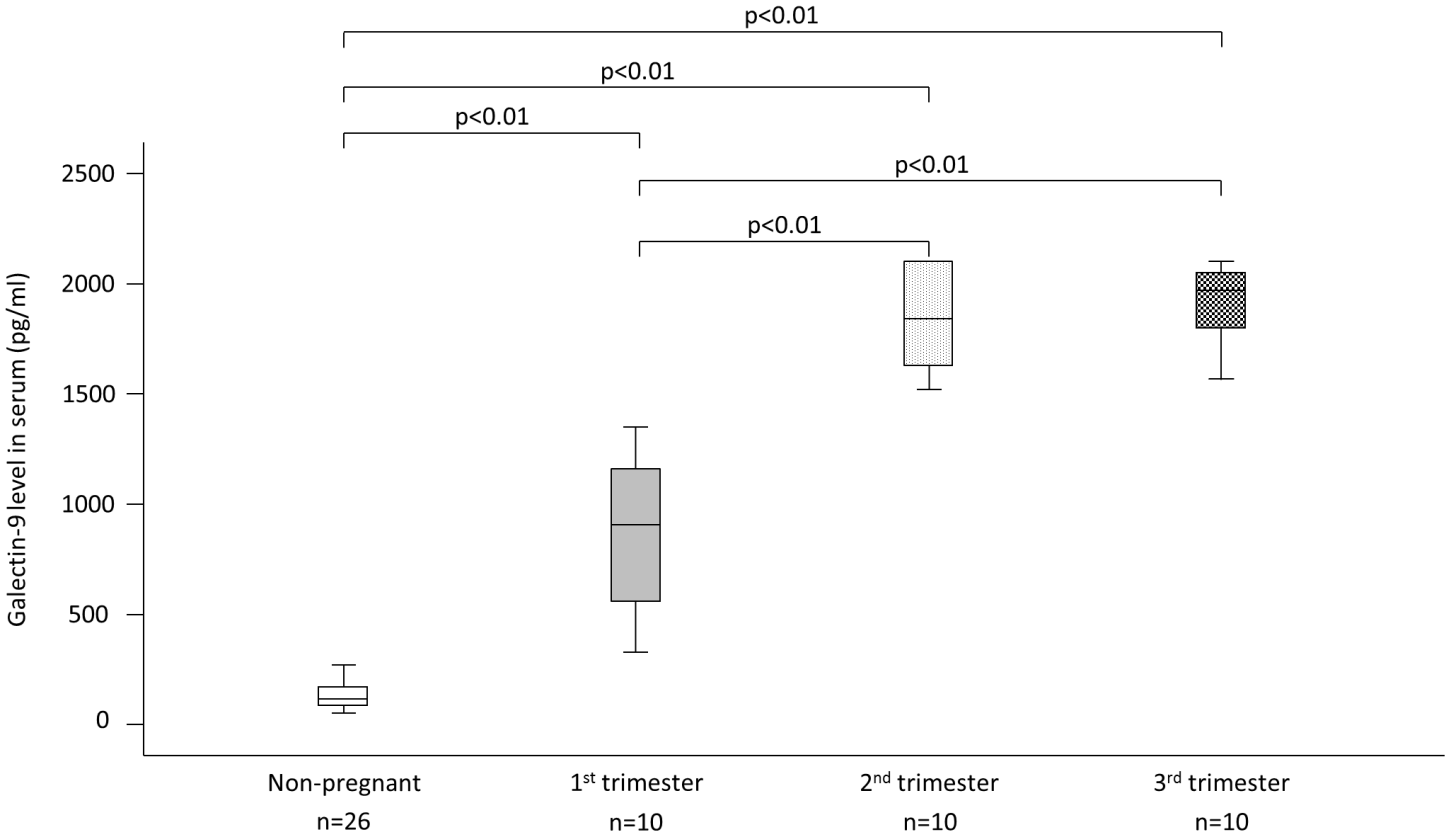
Circulating Galectin-9 levels throughout pregnancy and in non-pregnant women. Serum Galectin-9 levels of normal pregnant women during each trimester of pregnancy and in non-pregnant women. The solid bars represent medians; the boxes indicate the interquartile ranges and the lines show the most extreme observations. Differences were considered statistically significant for P-values ≤0.05.

Serum Galectin-9 levels differ significantly between non-pregnant and healthy pregnant women in each trimester (Fig. 4). Analyzing Gal-9 levels throughout pregnancy we found an increasing tendency with a significant elevation of serum Gal-9 concentration in the second and third trimester compared to the first trimester (Fig. 4).

## Discussion

The maternal immune system undergoes profound transformation as early as the beginning of pregnancy. These prominent changes are directed to protect the fetus from a detrimental immune response.

Widely distributed across the cells of innate and adaptive immune system and in nonmyeloid cell lineages, TIM family members are emerging as central modulators of numerous facets of the immune response. Much remains to be understood regarding the pleiotropic role of TIM-3 molecule in reproductive immunology.

Current thinking suggests that Galectin-9 is immunomodulatory to TIM-3 positive cells. This alters the production of cytokines, leads to the death of Th1 cells and selective loss of IFN-γ production by T cells, and furthermore influences the ability to induce T cell tolerance [17], [22].

We found that CD8+ T cells expressing TIM-3 produced significantly lower level of proinflammatory and Th17 cytokines compared to TIM-3 negative counterparts in the first and third trimester of pregnancy and in healthy non-pregnant controls, we suppose that Gal-9 inhibits proinflamamtory cytokine production through TIM-3 receptor activation downregulating Th1 and Th17 response. Based on our results we hypothesize that during pregnancy Gal-9 has the potential to control the cytokine production of TIM-3 positive CD8+ T cells favoring the establishment of Th2 dominance.

On the other hand we showed that the frequency and cytotoxic activity of CD8+ T cells expressing TIM-3 in the peripheral blood of pregnant women remained constant during the first and second trimester and their proinflammatory cytokine production was significantly decreased in the first trimester compared to TIM-3 negative counterparts. We suggest that TIM-3 expressing CD8+ T cells, while providing immune protection against invading pathogens, could be involved in the establishment and maintenance of healthy pregnancy via the production of cytokines during the first two trimesters.

In contrast to this observation TIM-3 positive CD8+ T cells in the third trimester were enriched in the peripheral blood compared to the first and second trimester. Although they produced less proinflammatory cytokines than TIM-3 negative CD8+ T cells they showed increased cytotoxic activity. These observations let us hypothesize that these cells could play a role in the physiological processes leading to the initiation of labor.

The systemic regulation of NK cells is essential for the achievement of successful reproductive outcome [23]. In line with many publications, peripheral blood NK cell levels were found not to be significantly changed throughout pregnancy, also as compared to non-pregnant controls [24].

NK cell parameters, either in absolute numbers or in proportion, subsets, functional activity such as cytotoxicity or secretory cytokine profile, receptor or gene expression, have been extensively investigated in peripheral, endometrial or decidual NK cells, but our study is the first to investigate TIM-3 expressing NK cells in peripheral blood prior to and throughout pregnancy.

We found that TIM-3 receptor expression by NK and cytotoxic NKdim cells was significantly increased in the third trimester when compared to the second trimester. In the case of NKbright cells we found no differences.

Investigating Th1, Th2 and Th17 cytokine production by NK cells and its subsets we did not find any characteristic differences between TIM-3 positive and negative counterparts in the case of Th2 and Th17 cytokines. Investigating Th1 cytokines we found that IL-2 production by TIM-3 positive CD56dim NK cells was significantly lower compared to TIM-3 negative CD56dim NK cells in the first and second trimester of pregnancy. IFN-γ production by TIM-3 positive CD56bright NK cells was significantly higher compared to TIM-3 negative CD56bright NK cells in the second trimester of pregnancy.

The interaction between soluble or membrane bound Gal-9 and TIM-3 positive NK cells could explain the impaired cytotoxic activity in the peripheral blood during the first and second trimester, although some publications suggest that this interaction is not only limited to TIM-3 and that Gal-9 can also act on NK cells independently of TIM-3 [25].

Several molecules modulate and influence the cells that are directly involved in the generation and maintenance of an active immunotolerance toward the fetus and accumulating evidence points to galectins, a family of endogenous glycan-binding proteins as an important regulator of pregnancy [26], [27].

Gal-9 has complex immunomodulatory roles involving effector cells of both innate and adaptive immunity [14], [26], [27]. This important pleiotropic modulator affects numerous immune cell types. In general, Gal-9 is thought to be involved in the activation of innate immune responses [28], [29] and the downregulation of Th1 and Th17 responses [15], [16], [18]. Although a number of recent papers have identified roles for Gal-9 in the inhibition of CD8+ T cells, expansion of regulatory T cells, regulation of NK cell function and promotion of Th2 cell migration, its role in mediating immunotolerance during pregnancy is only now emerging.

Gal-9 may regulate the immune function of NK cells and CD8+ T cells during pregnancy depending on the activation threshold, stage of pregnancy, inflammatory stimuli, and relative expression of cellular receptors.

The presence of Galectin-9 at the fetal-placental interface is already known [20], [21], [30], and our study demonstrates for the first time that Gal-9 is not only expressed by the placenta itself but also by regulatory immune cells (Gal-9+ Th cells) in the peripheral blood and it can be found at high concentration in the sera of healthy pregnant women compared to non-pregnant women. Analyzing serum Gal-9 levels throughout pregnancy we found an increasing tendency with a significant elevation in the second and third trimester compared to the first trimester, which correlates with the emergence of Gal-9+ Th cells in the peripheral blood of healthy pregnant women.

Since Oomizu et al. identified Gal-9+ Th cells in humans secreting large amount of Gal-9 upon stimulation and regulate Th17 development [18], we can suppose that this cell population could be one of the populations to secrete Gal-9 found in the sera of pregnant women.

A major unresolved issue is the exact role of Gal-9 in healthy pregnancy. Given the demonstration that serum Gal-9 levels are significantly elevated throughout pregnancy compared to non-pregnant women, it is tempting to hypothesize that one of the primary function of this molecule is to support healthy pregnancy suggesting that this physiological increase of Gal-9 during pregnancy is presumably a counter-regulatory mechanism aimed to regulate and control pro-inflammatory mechanisms during pregnancy.

Based on our result we can hypothesize that Gal-9 influences both CD8+ T and NK cells in the periphery of pregnant women but in different ways.

We demonstrated that serum Gal-9 level is increased during normal pregnancy, may result in decreased proinflammatory cytokine production by TIM-3 positive CD8+ T cells and an impaired cytotoxic activity by NK and NKdim cell expressing TIM-3. TIM-3 is known to be involved in tolerance induction [17] and now we can propose than TIM-3 cell surface molecule, widely expressed on innate and adaptive immune cells could control the immunoregulation during pregnancy.

Our results indicate that TIM-3 and Galectin-9 are able to initiate different functional outcomes in a cell-specific manner during pregnancy; however, further studies are needed to clarify the exact role of TIM-3/Gal-9 pathway at the feto-maternal site.
